# Supplementary material for: Multimorbidity Profile of COVID-19 Deaths in Portugal during 2020
Source: J Clin Med. 2022 Mar 29;11(7):1898. doi: 10.3390/jcm11071898 (PMC8999817; doi:10.3390/jcm11071898)
Supplement: Supplementary file 1 [file jcm-11-01898-s001.zip › jcm-1545206-supplementary.pdf]

Journal of Clinical Medicine

Multimorbidity Profile of COVID-19 Deaths in Portugal during 2020

Paulo Jorge Nogueira, Miguel de Araújo Nobre, Cecília Elias, Rodrigo Feteira-Santos, António Martinho, Catarina Camarinha Leonor Bacelar-Nicolau, Andreia Silva Costa, Cristina Furtado, Liliane Morais, Juan Rachadell, Mário Pereira Pinto, Fausto Pinto and António Vaz Carneiro

Corresponding author: Paulo Jorge Nogueira
Faculdade de Medicina da Universidade de Lisboa
Avenida Professor Egas Moniz
1649-028 Lisboa
pnogueira@medicina.ulisboa.pt

Supplementary Material

Figure S1: Weekly evolution of the Number of COVID-19 deaths, total (A) and by sex (B), and of new infected cases (C) in Portugal during 2020 1
Figure S2: Age distribution of COVID-19 deaths in Portugal during 2020 3
Figure S3: Distribution of COVID-19 number of deaths and COVID-19 death rates by health administrative region (A and B), district (C and D) and municipality of residence (E and F) 4
Figure S4: Geographical distribution of COVID-19 deaths by municipalities in absolute number and Crude Mortality Rates for total population (A and B), men (C and D), and women (E and F), Portugal, 2020 5

Table S1 Age distribution and comparison by characteristics 6
Table S2 Percentual presence of comorbidities (according to the Elixhauser Comorbidity Index) by Health Region of residence and by time-periods 8
Table S3 Percentage of Elixhauser multimorbidity levels and Elixhauser weighted multimorbidity indexes highest levels by time-periods 9

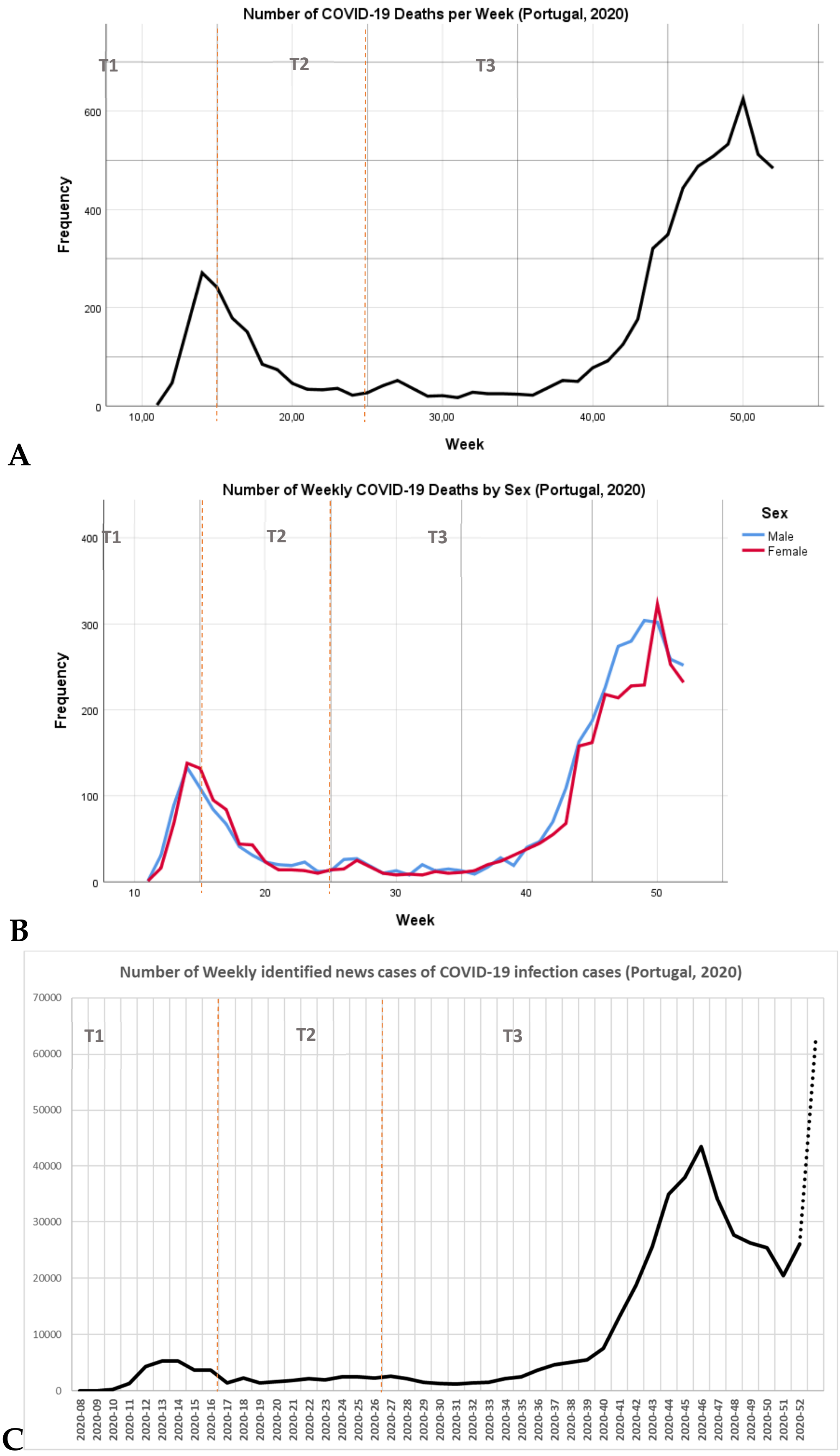

Figure S1. Weekly evolution of the Number of COVID-19 deaths, total (**A**) and by sex (**B**), and of new infected cases (**C**) in Portugal during 2020.

Notes: ISO Weeks numbers of 2020 are presented – week 53 is omitted because having only four days. **Abbreviations:** (T1) March 2 - June 2; (T2) June 3 - August 11; (T3) August 12 - December 31. Data source for (C): https://github.com/CSSEGISandData.

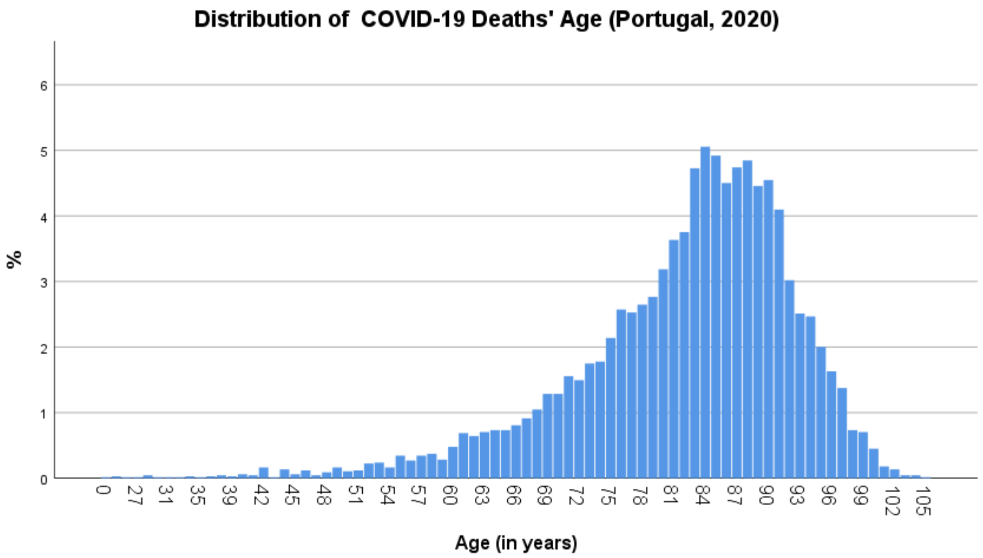

A

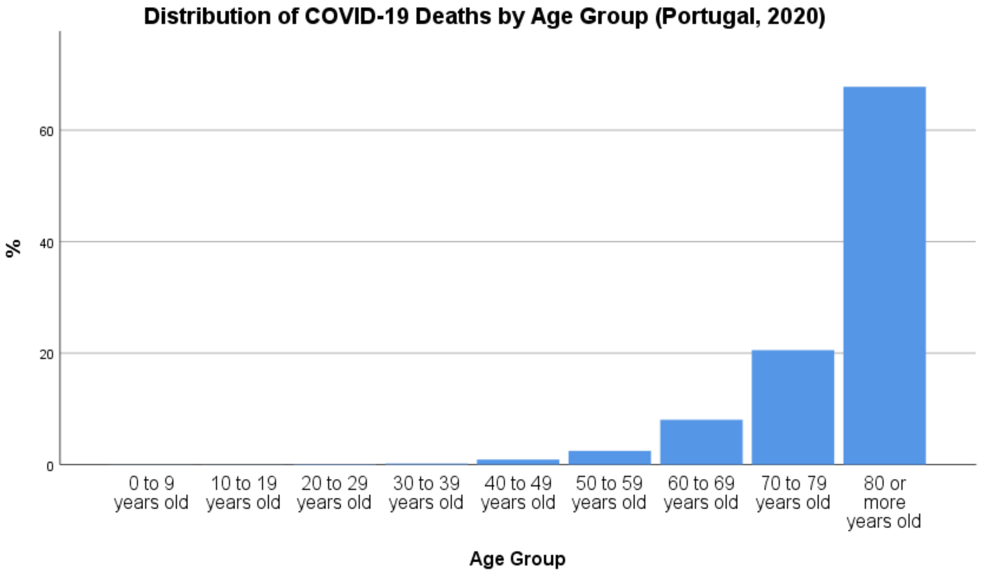

B

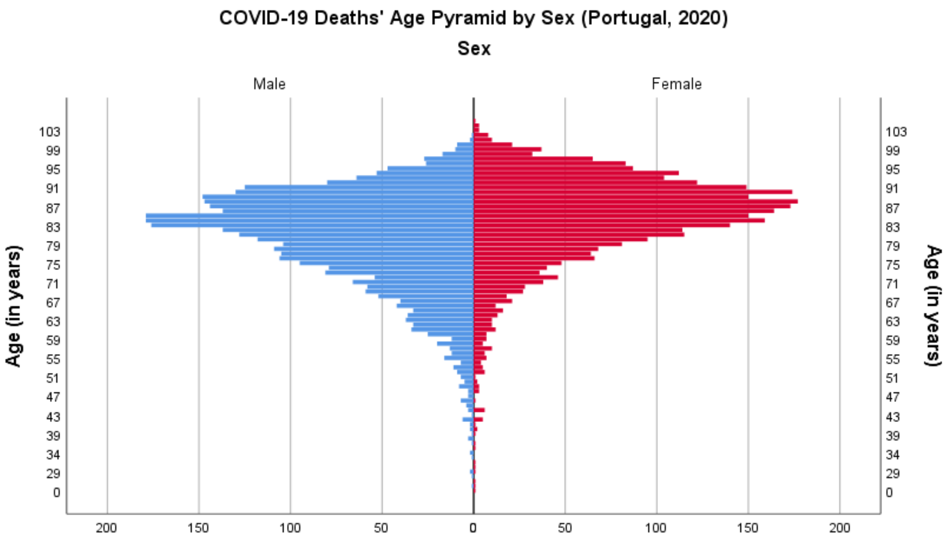

C

Figure S2. Age distribution of COVID-19 deaths in Portugal during 2020.

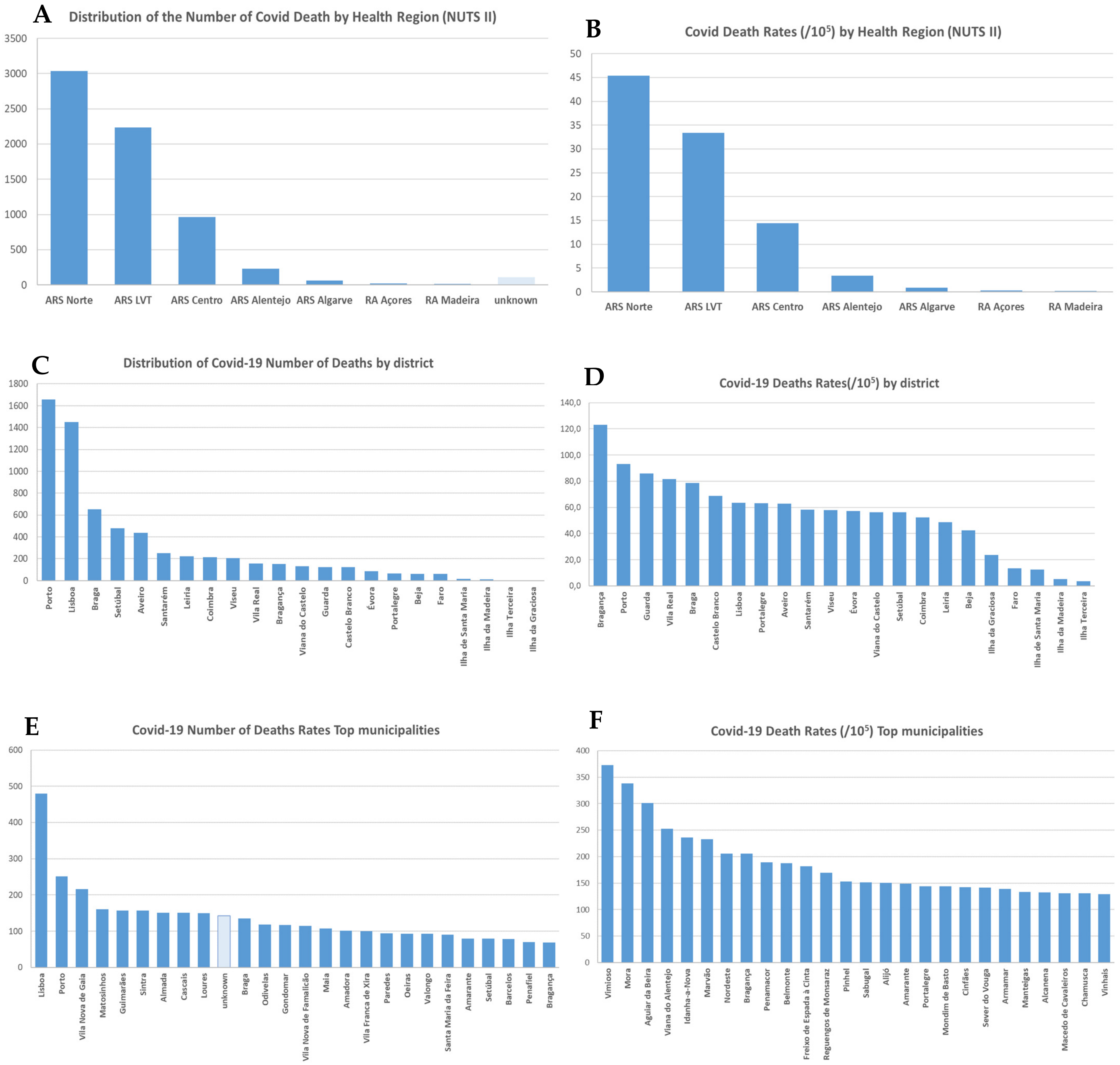

Figure S3. Distribution of COVID-19 number of deaths and COVID-19 death rates by health administrative region (**A**,**B**), district (**C**,**D**) and municipality of residence (**E**,**F**).

(A) Number of deaths by municipality

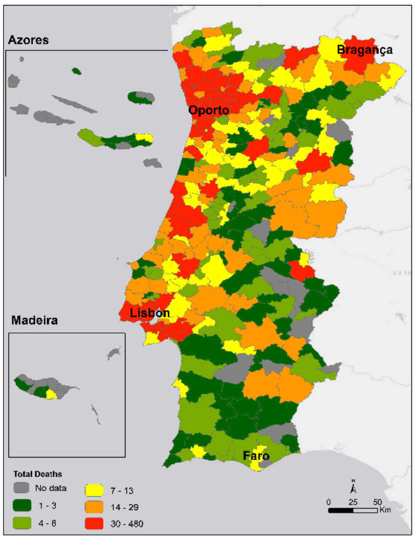

(B) CMR/100,000 inhab. by municipality

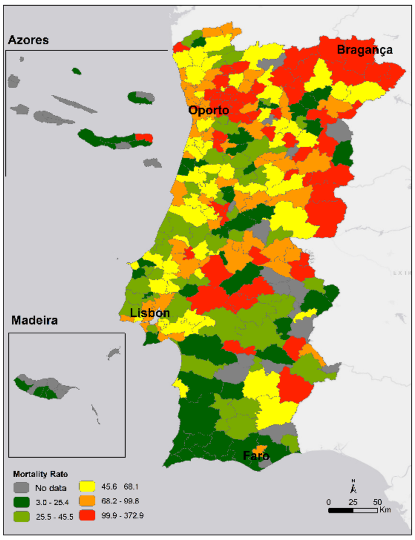

(C) Number of deaths by municipality - men

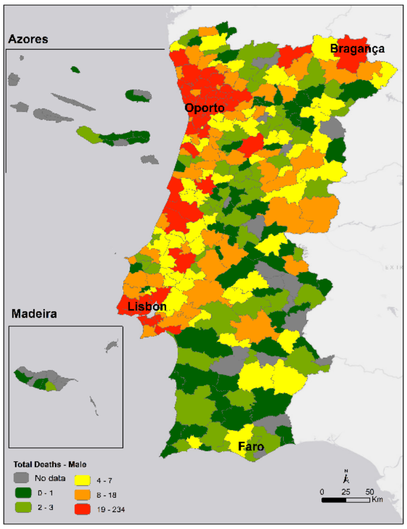

(D) CMR/100,000 men by municipality

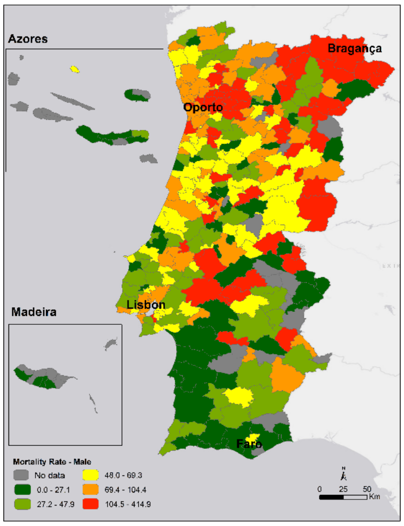

(E) Number of deaths by municipality - women

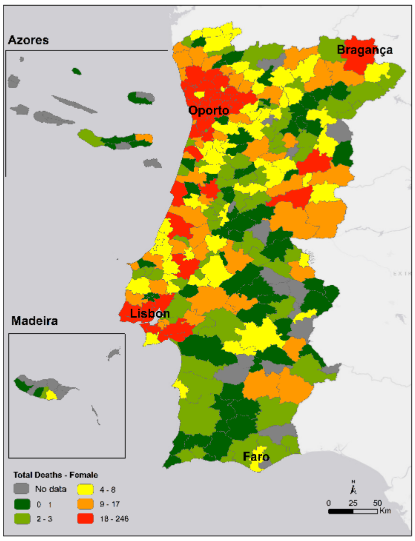

(F) CMR/100,000 women by municipality

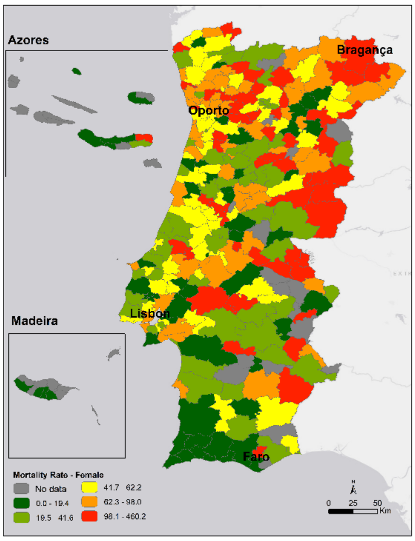

Figure S4. Geographical distribution of COVID-19 deaths by municipalities in absolute number and Crude Mortality Rates for total population (**A**,**B**), men (**C**,**D**), and women (**E**,**F**), Portugal, 2020.

Table S1. Age distribution and comparison by characteristics.

Age Distribution by Sex								
**Sex**	**Median**	**Mean**	**Standard Deviation**	**Min - Max**	**1st - 3rd Quartiles**	**n**	**Missing**	**p-Value**
**Female**	86	84.3	9.9	0 - 105	80 - 91	3192	7	**<0.001**
**Male**	82	79.9	10.7	19 - 102	74 - 88	3495	7	
**Age distribution by age group**								
**Age groups**	**Median**	**Mean**	**Standard deviation**	**Min - Max**	**1st - 3rd quartiles**	**n**	**Missing**	**p-value**
**0 to 9 years**	0	0	-	0 - 0	0 - 0	1	0	**<0.001**
**10 to 19 years**	19	19	0	19 – 19	19 – 19	2	0	
**20 to 29 years**	29	28	1.4	26 – 29	27 – 29	5	0	
**30 to 39 years**	37	35.5	3.1	30 - 39	34 – 38	13	0	
**40 to 49 years**	45	45	2.9	40 – 49	42 – 48	60	0	
**50 to 59 years**	56	55.4	2.6	50 – 59	53 – 58	165	0	
**60 to 69 years**	66	65.2	2.9	60 – 69	63 – 68	537	0	
**70 to 79 years**	76	75.2	2.8	70 – 79	73 – 78	1372	0	
**> 80 years**	87	87.8	4.9	80 – 105	84 – 91	4532	0	
**Age distribution per district**								
**Districts**	**Median**	**Mean**	**Standard deviation**	**Min - Max**	**1st - 3rd quartiles**	**n**	**Missing**	**p-value**
**Aveiro**	84	81	11.3	29 – 102	75 – 89	439	142	**<0.001**
**Beja**	87	85.2	9.7	45 – 99	81 – 92.2	60	142	
**Braga**	84	81.8	10.5	42 – 104	76 – 89	651	143	
**Bragança**	86	85.7	8.2	53 – 102	81 – 91	153	142	
**Castelo Branco**	87	85.7	9.1	49 – 105	81 – 92	122	142	
**Coimbra**	84.5	82.4	10.4	42 – 102	77 – 90	212	142	
**Évora**	86.5	84	9.8	42 – 98	81.2 – 90	86	143	
**Faro**	84	81	12.7	37 – 99	76.5 – 90	59	142	
**Guarda**	87	86	8.3	41 – 104	82 – 91	123	142	
**Leiria**	84	82.5	9.9	44 – 101	78 – 89	222	142	
**Lisboa**	84	81.6	11.3	0 – 102	76 – 89	1449	144	
**Portalegre**	85.5	84.4	8.6	61 – 102	80.2 – 90	66	142	
**Porto**	83	81	10.6	19 – 103	75 – 88	1650	151	
**Santarém**	84	83.6	9	46 – 104	79 – 90	250	142	
**Setúbal**	84	81.4	10.8	19 – 103	76 – 88	480	142	
**Viana do Castelo**	85	84.3	8.2	44 – 98	81 – 90	130	142	
**Vila Real**	84	83.2	9.3	55 – 99	77.5 – 90	155	143	
**Viseu**	85	83.9	10.4	31 – 100	80 – 90	205	142	
**Madeira Island**	87	85.6	8.9	64 – 97	80 – 91	13	142	
**Santa Maria Island**	84	80	13.8	48 – 99	73 – 90	17	142	
**Terceira Island**	80.5	80.5	12	72 – 89	76.2 – 84.8	2	142	
**Graciosa Island**	47	47	NA	47 – 47	47 – 47	1	142	
**Age distribution by health region**								
**Health region**	**Median**	**Mean**	**Standard deviation**	**Min - Max**	**1st - 3rd quartiles**	**n**	**Missing**	**p-value**
Unknown	83	80.7	10.1	49 - 101	75 - 88	142	0	**<0.001**
**ARS Alentejo**	87	84.7	9.3	42 - 103	81 - 90	227	1	
**ARS Algarve**	84	81.0	12.7	37 - 99	76.5 - 90	59	0	
**ARS Centro**	85	83.5	10.3	38 - 105	79 - 90	964	0	
**ARS LVT**	84	81.7	10.9	0 - 104	76 - 89	2234	2	

**ARS North**	84	81.7	10.9	19 - 104	76 - 89	3028	11	
**ARA Açores**	83	78.4	14.9	47 - 99	71.5 – 89.2	20	0	
**ARA Madeira**	87	85.6	8.9	64 - 97	80 - 91	13	0	

Age distribution by Death Place

Death Place	Median	Mean	Standard deviation	Min - Max	1st - 3rd quartiles	n	Missing	p-value
**Health Institution**	84	81.8	10.5	0 – 104	76 – 89	6079	4	**<0.001**
**At home**	85	82.6	12	37 – 101	79 – 90	277	9	
**Nursing home**	89	87.1	8.7	60 – 105	83 – 93	280	0	
**Private Health Institution**	80	80.6	6	70 – 90	77 – 84.5	11	0	
Unknown	87.5	84.2	12.8	50 – 101	80.8 – 93	40	1	

Pandemic period	Median	Mean	Standard deviation	Min - Max	1st - 3rd quartiles	n	Missing	p-value
**T1**	84	82.0	10.8	26 – 104	77 – 89	1320	71	0.481
**T2**	83	81.0	11.6	38 – 101	75 – 89	262	71	
**T3**	84	82.2	10.5	0 – 105	77 – 89	5046	69	

Age distribution by Basic Cause of deaths

Basic Cause	Median	Mean	Standard deviation	Min - Max	1st - 3rd quartiles	n	Missing	p-value
**U071 (laboratory confirmed)**	84	82.0	10.6	0 - 105	77 - 89	6598	12	0.803
**U072 (laboratory unconfirmed)**	85	81.2	11.8	38 - 99	79 - 88	89	2	

Notes: significant results are presented in bold font**. Abbreviations:** ARS, administrative region of health; RA, autonomous region. Periods: T1) March 2 - June 2; T2) June 3 - August 11; T3) August 12 - December 31.

Table S2. Percentual presence of comorbidities (according to the Elixhauser Comorbidity Index) by Health Region of residence and by time-periods.

Conditions	ARS Alentejo	ARS Algarve	ARS Centro	ARS LVT	ARS Norte	RA Azores	RA Madeira	P[a]	T1	T2	T3	P[a]
**Hypertension, uncomplicated**	25.44	34.43	27.67	30.13	30.88	10,00	61.54	0.068	28.59	31.82	30.22	0.537
**Diabetes, uncomplicated**	18.42	21.31	15.69	16.39	17.46	0,00	7.69	0.322	15.28	18.94	17.00	0.265
**Congestive heart failure**	9.65	14.75	13.78	15.24	14.51	10,00	15.38	0.501	14.75	11.74	14.53	0.487
**Renal failure**	10.53	9.84	13.68	12.95	12.74	5,00	15.38	0.765	13.31	11.74	12.78	0.782
**Cardiac arrhythmias**	7.89	16.39	10.97	10.35	10.82	10,00	7.69	0.600	10.59	7.95	10.74	0.399
**Chronic pulmonary disease**	6.58	8.20	6.84	7.14	7.22	5,00	15.38	0.944	6.73	6.82	7.25	0.804
**Solid tumor, without metastasis**	7.89	8.20	6.24	7.36	7.06	5,00	0.00	0.882	6.66	9.85	6.96	0.193
**Obesity**	7.02	9.84	4.02	4.67	5.84	0,00	7.69	0.076	4.77	4.92	5.37	0.676
**Other neurological disorders**	3.51	4.92	5.53	4.93	4.72	0,00	7.69	0.766	4.01	5.68	5.03	0.266
**Fluid and electrolyte disorders**	3.07	4.92	4.12	3.96	3.66	0,00	15.38	0.388	2.95	6.06	3.94	**0.043**
**Valvular disease**	2.19	6.56	2.92	3.08	3.18	0,00	7.69	0.577	3.10	1.14	3.21	0.176
**Peripheral vascular disorders**	3.07	1.64	1.91	1.98	2.15	0,00	7.69	0.725	1.82	1.52	2.18	0.579
**Pulmonary circulation disorders**	1.32	3.28	1.91	2.42	1.86	0,00	7.69	0.486	1.74	2.65	2.06	0.584
**Hypertension, complicated**	1.75	3.28	1.61	2.47	1.83	0,00	0.00	0.584	1.44	3.41	2.10	0.086
**Hypothyroidism**	3.51	1.64	1.21	2.20	1.67	0,00	0.00	0.245	1.59	3.03	1.82	0.285
**Metastatic cancer**	2.63	1.64	2.11	2.20	1.41	5,00	0.00	0.323	1.59	3.03	1.82	0.285
**Depression**	2.19	0.00	1.41	1.45	1.86	0,00	15.38	**0.007**	1.29	2.27	1.72	0.399
**Diabetes, complicated**	2.19	0.00	1.51	1.59	1.41	0,00	0.00	0.890	1.13	0.38	1.64	0.128
**Liver disease**	2.19	1.64	1.51	1.28	1.57	0,00	0.00	0.909	1.13	1.89	1.57	0.449
**Deficiency anaemia**	1.32	0.00	1.61	1.15	1.28	0,00	0.00	0.869	1.21	0.00	1.37	0.155
**Rheumatoid arthritis/collaged vascular disease**	0.88	0.00	0.70	1.32	1.35	5,00	0.00	0.413	1.21	0.76	1.21	0.805
**Lymphoma**	0.44	0.00	1.71	1.10	0.96	5,00	0.00	0.209	1.06	2.65	1.05	0.055
**Weight loss**	0.88	0.00	0.91	1.06	0.83	0,00	0.00	0.957	0.68	3.41	0.83	**<0.001**
**Coagulopathy**	0.44	0.00	1.21	0.93	0.58	0,00	0.00	0.455	0.61	1.14	0.81	0.604
**Alcohol abuse**	0.00	0.00	0.91	0.79	0.80	0,00	0.00	0.832	0.76	0.76	0.77	0.998

**Psychoses**	0.44	0.00	0.80	0.31	0.42	0,00	0.00	0.604	0.61	0.00	0.40	0.326
**AIDS/HIV**	0.00	0.00	0.30	0.22	0.48	0,00	0.00	0.681	0.15	2.27	0.30	**<0.001**
**Paralysis**	0.00	0.00	0.20	0.31	0.22	0,00	0.00	0.972	0.23	0.38	0.24	0.896
**Peptic ulcer disease, excluding bleeding**	0.00	0.00	0.30	0.04	0.19	0,00	0.00	0.651	0.23	0.00	0.12	0.529
**Drug abuse**	0.00	0.00	0.00	0.04	0.13	0,00	0.00	0.869	0.00	0.00	0.10	0.456
**Blood loss anaemia**	0.44	0.00	0.00	0.00	0.03	0,00	0.00	**0.036**	0.00	0.00	0.04	0.730

Notes: [a] Homogeneity test; significant results are presented in bold font. **Abbreviations:** ARS, administrative region of health; RA, autonomous region. Periods: T1) March 2 - June 2; T2) June 3 - August 11; T3) August 12 - December 31.

Table S3. Percentage of Elixhauser multimorbidity levels and Elixhauser weighted multimorbidity indexes highest levels by time-periods.

	Periods			
	**T1**	**T2**	**T3**	**p**
**Elixhauser score > 1**	38.1	42	26.6	<0.001
**Elixhauser score > 2**	20	20.1	14.5	<0.001
**Elixhauser score > 3**	8.3	9.1	6.1	0.004
**Elixhauser index ≥ 5**	3.2	4.2	2	0.004
**Elixhauser Windex AHRQ ≥ 5**	32.6	40.9	21.5	<0.001
**Elixhauser Windex VW ≥ 5**	34.8	39	23.2	<0.001

Notes: Elixhauser score > k – individuals with at least k+1 Elixhauser comorbidities identified in the death certificate. **Abbreviations:** T1 - March 2 - June 2; T2 - June 3 - August 11; T3 - August 12 - December 31.
